# CircGSAP regulates the cell cycle of pulmonary microvascular endothelial cells via the miR-942-5p sponge in pulmonary hypertension

**DOI:** 10.3389/fcell.2022.967708

**Published:** 2022-08-11

**Authors:** Yuanyuan Sun, Wenhui Wu, Qinhua Zhao, Rong Jiang, Jinling Li, Lan Wang, Shijin Xia, Mingjie Liu, Sugang Gong, Jinming Liu, Ping Yuan

**Affiliations:** ^1^ Department of Cardio-Pulmonary Circulation, Shanghai Pulmonary Hospital, School of Medicine, Tongji University, Shanghai, China; ^2^ Department of Respiratory and Critical Care Medicine, Shandong Provincial Hospital Affiliated to Shandong First Medical University, Jinan, China; ^3^ Department of Shanghai Institute of Geriatrics, Huadong Hospital, Fudan University, Shanghai, China; ^4^ Department of Lung Function, Shandong Provincial Hospital Affiliated to Shandong First Medical University, Jinan, China

**Keywords:** pulmonary hypertension second to chronic obstructive pulmonary disease, pulmonary microvascular endothelial cells, circGSAP, miR-942-5p, SMAD4

## Abstract

**Background** We recently demonstrated that circGSAP was diminished in lung tissues from patients with pulmonary arterial hypertension and in hypoxia-induced pulmonary microvascular endothelial cells (PMECs). However, the underlying role of circGSAP in PMECs remains unknown. The study aimed to investigate the contribution of circGSAP to proliferation, apoptosis and cell cycle of PMECs in hypoxic environment and explore the mechanism.

**Methods** The expression of circGSAP was quantified by real-time PCR or immunofluorescence in human lung tissue and PMECs. CircGSAP plasmid, circGSAP small interfering RNA (siRNA), miRNA inhibitor and target gene siRNA were synthesized to verify the role of circGSAP on regulating the proliferation, apoptosis, and cell cycle of PMECs.

**Results** CircGSAP levels were decreased in lungs and plasma of patients with pulmonary hypertension second to chronic obstructive pulmonary disease (COPD-PH) and were associated with poor outcomes of COPD-PH patients. Upregulation of circGSAP inhibited proliferation, apoptosis resistance and G1/S transition of PMECs. Dual luciferase reporter assays showed that circGSAP acted as a competitive endogenous RNA regulating miR-942-5p, and identified SMAD4 as a target gene of miR-942-5p, Then, we verified the functions of miR-942-5p and SMAD4 in PMECs. In addition, the effect of circGSAP siRNA on PMECs was mitigated by transfection of miR-942-5p inhibitor, and the effect of miR-942-5p inhibitor on PMECs was inhibited by SMAD4 siRNA.

**Conclusion** Our findings demonstrated that diminished circGSAP accelerated cell cycle to facilitate cell proliferation and apoptosis resistance through competitively binding miR-942-5p to modulate SMAD4 expressions in hypoxia-induced PMECs, indicating potential therapeutic strategies for PH.

## Introduction

Pulmonary hypertension (PH) is a frequent complication in patients with chronic obstructive pulmonary disease (COPD) (Singh et al., 2019; Gredic et al., 2021), and it is associated with increased risk for hospitalization, worse clinical course and increased mortality (Wells et al., 2012; Torres-Castro et al., 2021). The prevalence of PH in patients with COPD (COPD-PH) is not negligible; it has been reported to range between 23 and 91%, depending on the diagnostic criteria used to define it and the severity of the disease (Gredic et al., 2021; Torres-Castro et al., 2021). Interestingly, the vascular lesions occurring in COPD-PH patients are morphologically similar to idiopathic pulmonary arterial hypertension (IPAH) (Seeger et al., 2013; Nathan et al., 2019), this is accompanied by pulmonary microvascular endothelial cells (PMECs) dysfunction and loss of PMECs integrity, leading to proliferation and apoptosis resistance in the adventitia and smooth muscle media, and the formation of a neointima (Deng et al., 2015). Despite substantial improvements over the past decades in the diagnosis and treatment of COPD-PH, patients with COPD-PH still have a poor prognosis (Waxman et al., 2021). Therefore, novel therapeutic targets PMECs are urgently needed for treating COPD-PH, particularly in PMECs.

Circular RNAs (circRNAs) are non-coding RNAs that are widely found in mammalian cells and have many important regulatory functions at the posttranscriptional level (Hansen et al., 2013; Wilusz and Sharp, 2013; Lyu and Huang, 2017), dysregulated circRNAs play a role in the pathobiology of PH (Ma et al., 2020; Zhang et al., 2020; Jiang et al., 2021). Studies have shown that circCalm4 plays an important role in regulating pyroptosis of pulmonary artery smooth muscle cells (PASMCs) *via* the miR-124-3p/PDCD6 axis (Jiang et al., 2021). In addition, mmu_circ_0000790 was reported as a critical regulator in the viability and migration of hypoxic PASMCs through the miR-374c/FOXC1/Notch axis (Yang et al., 2020). Several studies have focused on the effects of circRNAs on PASMCs in PH, but few reports show a direct correlation between circRNAs in PMECs and PH.

In our previous study, we demonstrated that downregulation of circGSAP was linked to the occurrence and poor outcomes in patients with IPAH, which indicated that circGSAP could be an emerging biomarker for diagnosis and prognosis in IPAH (Yuan et al., 2021). However, the underlying mechanisms of circGSAP function in COPD-PH patients and PMECs remain largely unknown. Therefore, the present study aimed to comprehensively investigate the mechanism underlying circGSAP action on PMECs *via* miRNA sponge, which may reveal novel therapeutic targets in COPD-PH.

## Matertials and methods

### Clinical samples

Human plasma samples from 42 incident patients with COPD-PH and 43 healthy individuals were obtained from the Shanghai Pulmonary Hospital, School of Medicine, Tongji University, from January 2015 to October 2019.

Lung tissues from four COPD-PH who treated with lung transplantation and four healthy subjects were obtained from the Shanghai Pulmonary Hospital, School of Medicine, Tongji University, in the period from 1 January 2019 to 30 October 2021. The diagnosis of PH was established for the European Society of Cardiology and the European Respiratory Society guidelines (Galie et al., 2016). Patients with other PH classifications were excluded. The study was approved and supervised by the Ethics Committee of Shanghai Pulmonary Hospital (number: K20-150Y). Written informed consent was obtained from all subjects.

### Mammalian cell lines

Briefly, all primary human PMEC lines were purchased from Science Cell (BK-3000, Shanghai, China) and cultured in endothelial cell medium (ECM, BK-1001, Shanghai, China) in a CO_2_ (5%) atmosphere at 37°C. Cells in passages three to eight were selected for subsequent experimentation. For hypoxic culture, PMECs were exposed to CO_2_ (5%)/O_2_ (5%)/balance of N_2_ for 24 h.

### Plasmid, siRNA and cell transfection

The circGSAP cDNA was amplified and cloned into a circRNA overexpression vector pLC5-ciR (Geneseed, Guangzhou, China) using the restriction enzymes EcoRI and BamHI, and the sequence was confirmed. Three siRNAs targeting circGSAP and SMAD family member 4 (*SMAD4)* were designed and synthesized by GenePharma (Shanghai, China). For circGSAP overexpression and silencing or *SMAD4* silencing, PMECs were transfected with the overexpression plasmid or siRNA using Lipo2000 (11668019, Invitrogen, United States).

For miR-942-5p overexpression and inhibition, PMECs were transfected with 40 nM miRNA mimics (miR-942-5p) or 80 nM miRNA inhibitor (miR-942-5p inhibitor) (GenePharma, Shanghai, China) using Lipo2000. Control groups were treated with equal concentrations of non-targeting mimics or negative control sequences to control for nonspecific effects. Primers used for cloning, the sequences of siRNAs, miRNA mimics and inhibitors are all listed in Supplementary Table S1.

### Cell cycle analysis

Cells in the G0/G1, S, and G2/M phases were detected using flow cytometry. Briefly, cells were harvested by trypsinization and fixed with 70% ethanol at 4°C. The fixed cells were centrifuged at 1000 × g for 5 min and resuspended in 500 μl staining buffer before detection. Ten microliters RNase A was added, mixed, and 25 μl propidium iodide (C1052, Beyotime, China) was added. The samples were then incubated in a 37°C water bath for 30 min. Finally, the cells were filtered through a 400 mesh sieve and detected using flow cytometry.

### Cell proliferation assay

Cells (2 × 10^3^) were seeded onto 96-well dishes in ECM and maintained at 37°C. A Cell Counting Kit-8 (CCK-8, CK04, Dojindo, Japan) was used to measure cell proliferation at different time points. The relative level was then obtained by normalization of data from at least three independent experiments.

### 5-Bromo-2′-Deoxyuridine (BrdU) assay

DNA replication was evaluated using the BrdU assay. Cells (2 × 10^3^) were seeded onto 96-well dishes in ECM and maintained at 37°C. A BrdU Assay (6813, Cell Signaling Technology, United States) was performed the assay following the manufacturer’s instructions. BrdU incorporation was measured at 450 relative light unit (RLU) with the Varioskan Flash (Thermo Scientific, United States).

### EdU cell proliferation kit

Cell proliferation assay was performed using the EdU Cell Proliferation Kit with Alexa Fluor 488 (CX002, Epizyme, China). Briefly, the cells were seeded in 96-well plates at a density of 5 × 10^3^ cells/well for 48 h after transfection. Then, the cells were incubated with 10 μM EdU for 2 h at 37°C. After being fixed with 4% paraformaldehyde for 15 min, the cells were treated with 0.3% Triton X-100 for 10 min and rinsed with wash buffer three times. Thereafter, the cells were exposed to 50 µl of click reaction cocktail for 30 min and then incubated with 1 × Hoechst 33342 to stain the cell nuclei for 10 min. Images were captured using a microscope. The percentage of EdU-positive cells was defined as the proliferation rate. All the experiments were performed in triplicate.

### Calcein-AM/PI staining

PMECs were cultured on 24-well plates (2 × 10^5^ cells) for 24 h. Then, fluorescence was assessed after calcein-AM/PI staining (Calcein-AM/PI Double Stain Kit, 40747ES76, YEASEN, China). Experiments were conducted according to the manufacturer’s instructions. For each sample, 5 µl (2 µM) of calcein-AM and 5 µl (2 µM) of PI were added and incubated for 30 min in an incubator (37°C) in the dark. Then, the cells were imaged using a fluorescence microscope.

### TUNEL assay

About 5 × 10^6^ cells/ml were fixed using 4% paraformaldehyde at room temperature for 30 min, to which PBS containing 0.3% Triton X-100 was further added. A TUNEL detection solution was prepared based on the instructions in the TUNEL cell apoptosis detection kit (Dead End Fluorimetric Kit, G3250, Promega, United States). The samples were then observed under a fluorescence microscope, with an excitation wavelength ranging from 450 to 500 nm and an emission wavelength for detection from 515 to 565 nm.

### Dual luciferase activity reporter system

To elucidate circRNA-miRNA-mRNA interaction, a circGSAP/SMAD4 sequence containing the putative target sites for miR-942-5p was synthesized and cloned into the pGL3-promoter downstream of firefly luciferase (circGSAP-wt/SMAD4-wt). Mutant circGSAP/SMAD4 (circGSAP-mut/SMAD4-mut) was also generated with mutant of the complementary sites. After co-transfection of the reporter vector and miR-942-5p mimics or negative control into 293T cells, firefly luciferase activity was measured using a dual-luciferase reporter assay kit (E1960, Promega, United States) against that of Renilla luciferase. Each assay was repeated for five independent experiments. Primers and oligonucleotide sequences are listed in Supplementary Table S1.

### Fluorescence *in Situ* hybridization (FISH)

A fluorescence *in situ* hybridization (FISH) assay was performed in PMECs. Cy3-labeled circGSAP probe was designed by GeneSeed (Guangzhou, China). The signals of the probes were detected by a FISH Kit (GeneSeed Guangzhou, China) according to the manufacturer’s instructions. Samples were fixed in 4% paraformaldehyde and then incubated with Cy3-labeled circGSAP probe. Mix overnight at 42°C. The images were analyzed with a fluorescence microscope. The sequences of the detection probes are listed in Supplementary Table S1.

### Reverse transcription and quantitative real-time PCR (qRT-PCR)

Total RNA was extracted from cultured PMECs and lung tissue using TRIZOL (15596018, Invitrogen, United States). RNA samples in the medium were extracted and isolated from PMECs medium per the manufacturer’s protocol. Cytoplasmic and nuclear RNA was isolated using a PARIS^™^ kit (AM1921, Thermo Fisher Scientific, United States), according to the manufacturer’s protocol. For each sample, 1000 ng of total RNA was converted to cDNA using a Superscript First-Strand cDNA Synthesis Kit (K1612, Invitrogen, United States). qRT-PCR was carried out (Roche, Germany) with SYBR Green I (902905, Applied Biosystems, United States). The threshold cycle (CT) was determined and relative mRNA and miRNA levels were calculated based on the CT values. The data were analyzed using the 2^−ΔΔCT^ method. The data of each sample were normalized to GAPDH or U6 mRNA data. The key primers are listed in Supplementary Table S1.

### Western blot

PMECs were washed with ice-cold PBS and protein was extracted using RIPA buffer (P0013B, Beyotime Technology, China) according to the manufacturer’s instructions. The extracted proteins were centrifuged at 15,000 *g* for 15 min at 4°C and the protein concentrations were determined using the bicinchoninic acid protein (BCA) assay (23250, Pierce Chemical, United States). Protein lysates were electrophoretically separated on SDS-PAGE and transferred to PVDF membranes. The membranes were blocked in 5% milk and incubated with β-actin antibody (8H10D10-3700, Cell Signaling Technology, diluted 1:1000), CKD6 antibody (DCS83-3136, Cell Signaling Technology, diluted 1:2000) and SMAD4 antibody (D3R4N-4635, Cell Signaling Technology, diluted 1:1000) overnight at 4°C. The filters were washed in 0.25% Tween 20–TBS and incubated with secondary antibodies diluted 1:5000 in 5% milk and then washed again. The signals were analyzed using Image Lab software (Bio-Rad, Hercules, CA, United States).

### Statistical analysis

Statistical analysis was done using GraphPad Prism 8. The data are presented as mean ± SE. As indicated, Student’s unpaired *t*-test (two-tailed) was used to compare continuous values between two groups. One way ANOVA was used to compare continuous values between multiple groups. A *p*-value of less than 0.05 was considered statistically significant. For each experiment, data are representative of at least three replicates with similar results.

## Results

### CircGSAP is downregulated in patients with COPD-PH, and is associated with occurrence and poor outcomes

**FIGURE 1:**
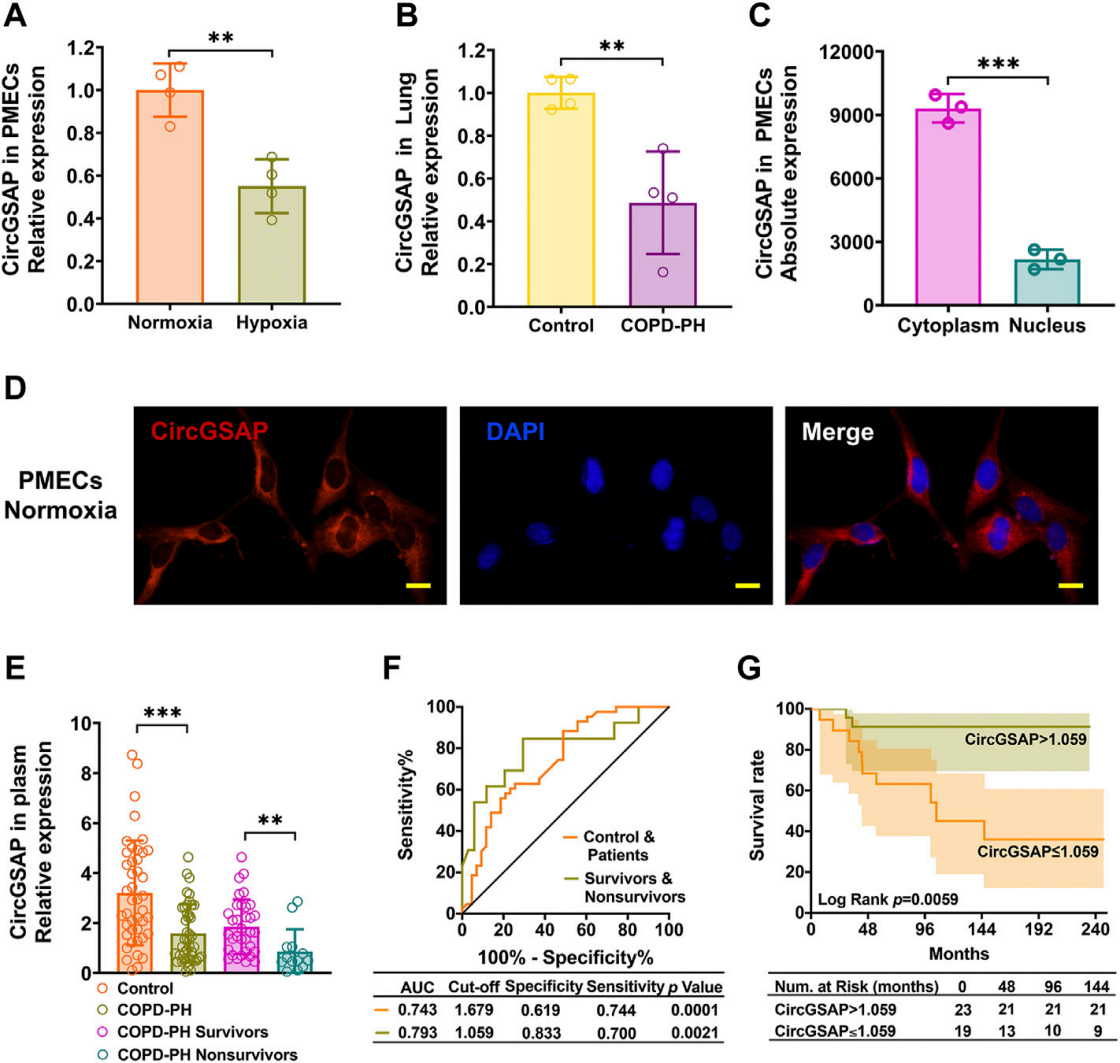
The expression of circGSAP in human PMECs, lung tissues and plasma. **(A)** Quantitative reverse transcription PCR (qRT-PCR) for the abundance of circGSAP in normoxia and hypoxia of PMECs (*n* = 4). **(B)** Expression levels of circGSAP in lung tissues of COPD-PH were determined by qRT-PCR (*n* = 4). **(C)** Levels of circGSAP in the cytoplasmic and nuclear fractions of PMECs (*n* = 3). **(D)** FISH was used to determine the distribution of circGSAP in PMECs. **(E)** Expression levels of circGSAP in plasma samples of COPD-PH, survivors and nonsurvivors were determined by qRT-PCR. **(F)** ROC curves of circGSAP in patients with COPD-PH, survivors and nonsurvivors with COPD-PH. **(G)** Kaplan-Meier survival analysis for mortality stratified by the cutoff values of circGSAP in COPD-PH. All data are presented as the mean ± SEM. (scale bar, 10 μm). *p < 0.05; **p < 0.01; ***p < 0.001.

CircGSAP was generated from exon 15 to exon 19 of the gamma-secretase activating protein (*GSAP*) (Supplementary Figure S1A). When comparing the expression of circGSAP in normoxia and hypoxia PMECs, we found hypoxia significantly induced the downregulation of circGSAP levels (Figure 1A). In human lung tissue from COPD-PH patients, the expression of circGSAP was also highly decreased compared to that of healthy subjects (Figure 1B). To investigate the cellular location of circGSAP, we performed qRT-PCR following nuclear/cytoplasmic fractionation and found circGSAP was mostly distributed in the cytoplasm of PMECs (Figure 1C). Then, a junction-specific probe for circGSAP was used in FISH, revealing that circGSAP mainly expressed in the cytoplasm of PMECs (Figure 1D).

We performed qRT-PCR analyses on plasma samples from 42 patients with COPD-PH and 43 plasma samples from healthy individuals. The baseline characteristics of them are shown in Table 1. No patient was lost to follow-up during 72.4 ± 48.3 months. The expression of plasma circGSAP was significantly lower in patients with COPD-PH than the control subjects, and dramatic lower in patients with nonsurvivors than survivors (Figure 1E). Next, we constructed receiver operating characteristic (ROC) curves to assess the circGSAP predictive value. The areas under the curve (AUCs) for patients with COPD-PH were 0.743 and for nonsurvivors with COPD-PH were 0.793 (Figure 1F), indicating that circGSAP might be a promising diagnostic and prognostic indicator for COPD-PH. Patients with COPD-PH with low plasma circGSAP levels had poorer outcomes (Figure 1G).

**TABLE 1:** Baseline characterristics in patients with COPD-PH and healthy controls.

Characteristics	COPD-PH patients (*n* = 42)	Control subjects (*n* = 43)
Age, years	64. 1 ± 10.6	53.4 ± 18.5	
Male/Female, *n*	26/16	19/24	
Nonsurvivors, *n*	12	0	
BMI, kg/m^2^	23.2 ± 2.1	—	
NT-proBNP, pg/ml	471 (160–1575)	—	
WHO-FC III/IV, *n* (%)	22.0 ± 3.6	—	
6MWD, m	312.4 ± 142.3	—	
mRAP, mmHg	3.2 ± 2.3	—	
mPAP, mmHg	31.1 ± 12.6	—	
mPAWP, mmHg	9.5 ± 6.7	—	
PVR, Wood units	4.2 ± 2.7	—	
CO, L/min	5.3 ± 1.3	—	
Specific therapy, *n* (%)			
PDE-5 inhibitors	2 (4.8)	—	
ERAs	—	—	
sGC stimulator	—	—	
Combination	—	—	

6MWD, 6-min walk distance; BMI, body mass index; CO, cardiac output; ERA, Endothelin receptor antagonist; HR, heart rate; mPAP, mean pulmonary arterial pressure; mPAWP, mean pulmonary capillary wedge pressure; mRAP, mean right atrial pressure; PDE-5, phosphodiesterase type 5; PVR, pulmonary vascular resistance; sGC, solube guanylate cyclase; WHO-FC, World Health Organization Functional Class.

### CircGSAP inhibited the over-proliferation, G1/S transition and promoted apoptosis of PMECs

**FIGURE 2:**
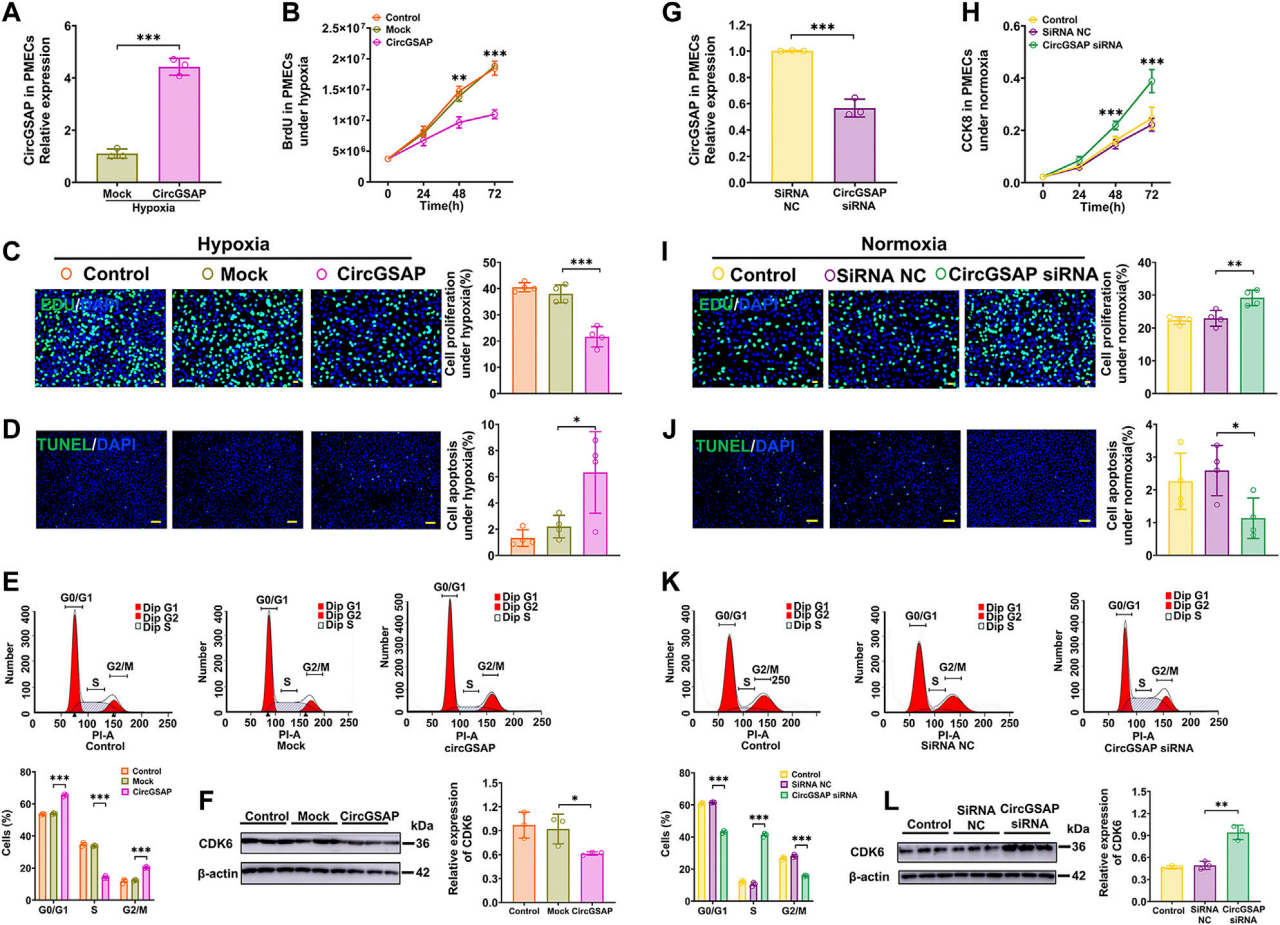
Effects of circGSAP on the proliferation, apoptosis and cell cycle of PMECs. **(A)** Expression levels of circGSAP in PMECs transfection with circGSAP plasmid (*n* = 3). **(B**–**E)** Cell proliferation analysis, EDU analysis, cell apoptosis analysis (*n* = 4) and cell cycle analysis (*n* = 3) of PMECs with overexpressing circGSAP under hypoxia. **(F)** The expression of CDK6 in PMECs transfected with circGSAP was analyzed by WB (*n* = 3). **(G)** Expression levels of circGSAP in PMECs after transduction with circGSAP siRNA (*n* = 3). **(H**–**K)** Cell proliferation analysis, EDU analysis, cell apoptosis analysis (*n* = 4) and cell cycle analysis (*n* = 3) of PMECs with silencing circGSAP under normoxia. **(L)** The expression of CDK6 in PMECs transfected with circGSAP siRNA was analyzed by WB (*n* = 3). All data are presented as the mean ± SEM. (scale bar, 100 μm). *p < 0.05; **p < 0.01; ***p < 0.001.

To further study the potential function of circGSAP, we successfully overexpressed circGSAP in PMECs *via* circGSAP plasmid transfection (Figure 2A). CircGSAP overexpression significantly reduced the proliferation of PMECs under hypoxia, as assessed by BrdU assay and EdU labeling (Figures 2B,C). TUNEL staining showed that circGSAP promoted apoptosis of PMECs (Figure 2D). Cell cycle analysis showed that circGSAP inhibited the G1/S transition of PMECs (Figure 2E). Cyclin-dependent kinase 6 (CDK6), as a protein marker of G1/S transition, was inhibited expression with overexpression circGSAP in PMECs (Figure 2F). Under normoxic conditions, overexpression of circGSAP also inhibited proliferation, G1/S transition, and apoptosis resistance of PMECs, as shown in Supplementary Figure S2. On the other hand, we designed small interfering RNA (siRNA) of circGSAP (circGSAP siRNA) that could efficiently diminish the expression of circGSAP in PMECs under normoxic conditions (Figure 2G). And silencing circGSAP significantly resulted in proliferation of PMECs (Figures 2H,I), reduced their apoptosis (Figure 2J), changed the cell cycle (Figure 2K) and enhanced the expression of CDK6 (Figure 2L). These results suggest that circGSAP is an essential and sufficient player in regulating the proliferation, apoptosis, and cell cycle of PMECs.

### CircGSAP adsorbs miR-942-5p and negatively regulates its expression

**FIGURE 3:**
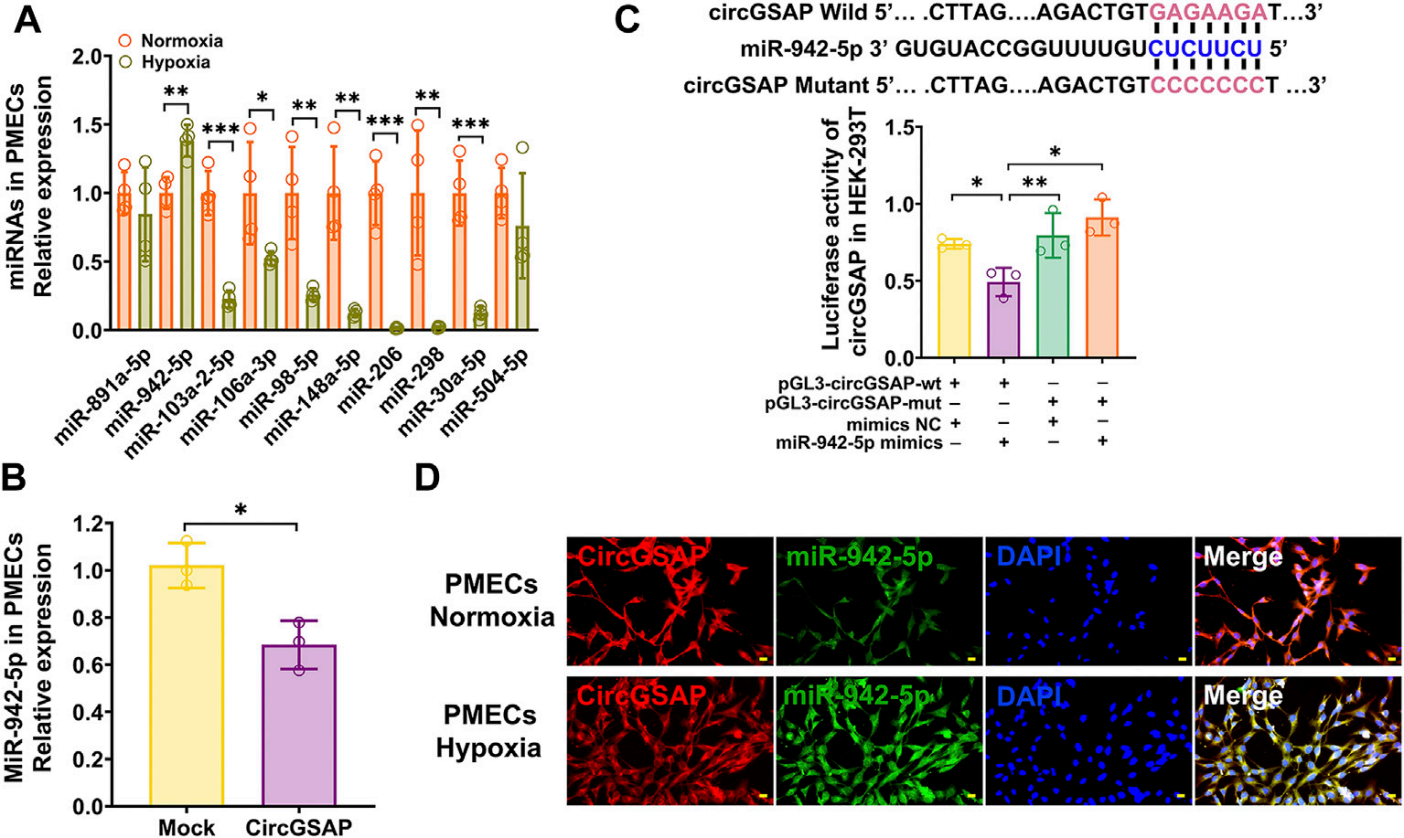
CircGSAP acts as efficient miR-942-5p sponge. **(A)** Bioinformatics websites predicted circGSAP with associated 10 miRNAs, and qRT-PCR was used to detect miRNAs expression levels in hypoxia treated PMECs (*n* = 3). **(B)** Expression levels of miR-942-5p in PMECs with transfecting circGSAP were determined by qRT-PCR (*n* = 3). **(C)** TargetScan predicted binding sites of circGSAP and miR-942-5p, and dual luciferase assays were used to validate the interactions between circGSAP and miR-942-5p (*n* = 3). **(D)** FISH was used to observe the colocalization of circGSAP and miR-942-5p in the cytoplasm of PMECs. All data are presented as the mean ± SEM. (scale bar, 20 μm). *p < 0.05; **p < 0.01; ***p < 0.001.

We further investigated the downstream targets of circGSAP in PMECs. Because circGSAP is mainly expressed in the cytoplasm of PMECs (Figure 1C), in which, circRNAs can repress the function of miRNAs by binding them as competing endogenous RNAs (Hansen et al., 2013; Kristensen et al., 2019). Using TargetScan, miRanda and RNAhybrid databases, we putative several miRNA targets that potentially interact with circGSAP, such as miR-891a-5p, miR-942-5p, miR-103a-2-5p, miR-106a-3p, miR-98-5p, miR-148a-5p, miR-206, miR-298, miR-30a-5p and miR-504-5p (Figure 3A). In those miRNAs, qRT-PCR showed that miR-942-5p was upregulated in hypoxia-induced PMECs, and diminished after overexpression of circGSAP levels (Figures 3A,B), which is consistent with the miRNA acts as circRNA sponge to influence the occurrence and development of diseases. and miR-942-5p regulates cell proliferation had been reported (Li et al., 2020; Wang et al., 2020; Gu et al., 2021; Zhang et al., 2021), so, we speculate that miR-942-5p may be downstream of circGSAP, which affecting the function of PMECs. Then, potential binding sites between circGSAP and miR-942-5p were predicted using Circular RNA Interactome, and the representative putative target sites of miR-942-5p are shown in Figure 3C. To validate whether circGSAP indeed acts as miR-942-5p sponge in PMECs, dual luciferase reporter assays were performed. The firefly luciferase reporter activity was significantly decreased in the miR-942-5p mimics and the circGSAP wild-type (wt) transfection group, whereas the circGSAP mutant (mut) group showed no notable changes in luciferase reporter activity (Figure 3C). Then we used a FISH assay, and colocalization between circGSAP and miR-942-5p was observed in the cytoplasm of PMECs under both normoxic and hypoxic conditions (Figure 3D). These results indicate that circGSAP serves as a sponge for miR-942-5p in PMECs.

### MiR-942-5p inhibitors reversed the effects of circGSAP siRNA on PMECs proliferation, apoptosis and cell cycle

**FIGURE 4:**
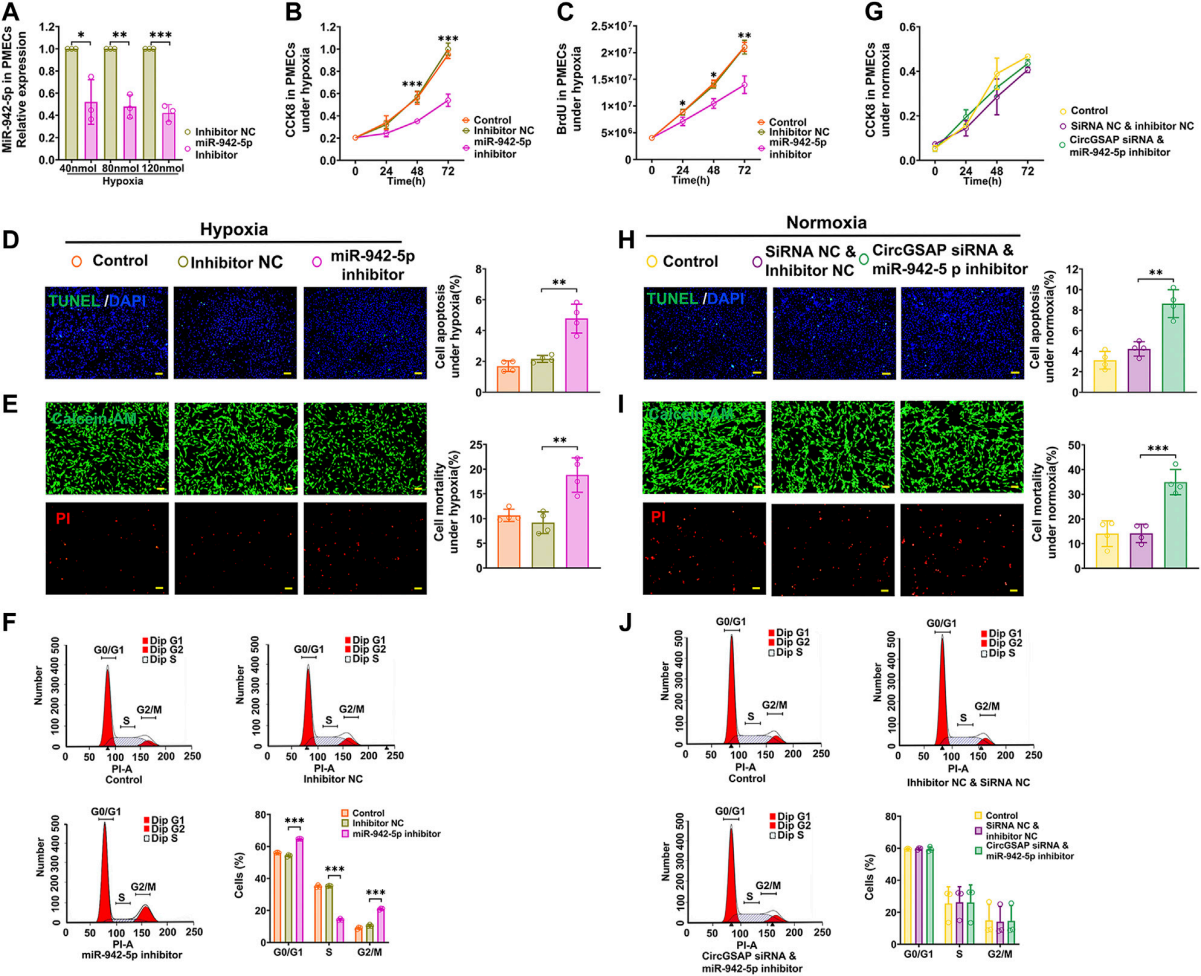
MiR-942-5p inhibitors reversed the effects of circGSAP siRNA on PMECs proliferation, apoptosis and cell cycle. **(A)** Expression levels of miR-942-5p in PMECs treated with miR-942-5p inhibitor under hypoxia (*n* = 3). **(B**–**F)** Cell proliferation analysis, apoptosis analysis, mortality analysis (*n* = 4) and cell cycle analysis (*n* = 3) of PMECs with miR-942-5p inhibitor under hypoxia. **(G**–**J)** Cell proliferation analysis, apoptosis analysis, mortality analysis (*n* = 4) and cell cycle analysis (*n* = 3) of PMECs with co-transfecting circGSAP siRNA and miR-942-5p inhibitor under normoxia. All data are presented as the mean ± SEM. (scale bar, 100 μm). *p < 0.05; **p < 0.01; ***p < 0.001.

MiR-942-5p has never been studied in PMECs, so we used a miR-942-5p inhibitor to examine its role on proliferation, apoptosis and cell cycle of PMECs. The level of miR-942-5p was significantly downregulated by its inhibitor (Figure 4A), associated with a clearly inhibition of proliferation measured by CCK8 and BrdU assay in PMECs exposed to hypoxia (Figures 4B,C). Meanwhile, apoptosis of PMECs was promoted (Figures 4D,E) and G1/S transition was blocked in PMECs (Figure 4F). These results were also observed in PMECs under normoxic conditions (Supplementary Figure S3). We then assayed cellular proliferation, apoptosis and cell cycle after co-transfection of circGSAP siRNA and miR-942-5p inhibitor in PMECs, and found over-proliferation, apoptosis resistance and G1/S transition were all significantly reversed in this condition (Figures 4G–J). Taken together, these results demonstrate that circGSAP suppressed proliferation, G1/S transition and promoted apoptosis in PMECs by inhibiting miR-942-5p.

### SMAD4 is the target of miR-942-5p and is regulated by circGSAP

**FIGURE 5:**
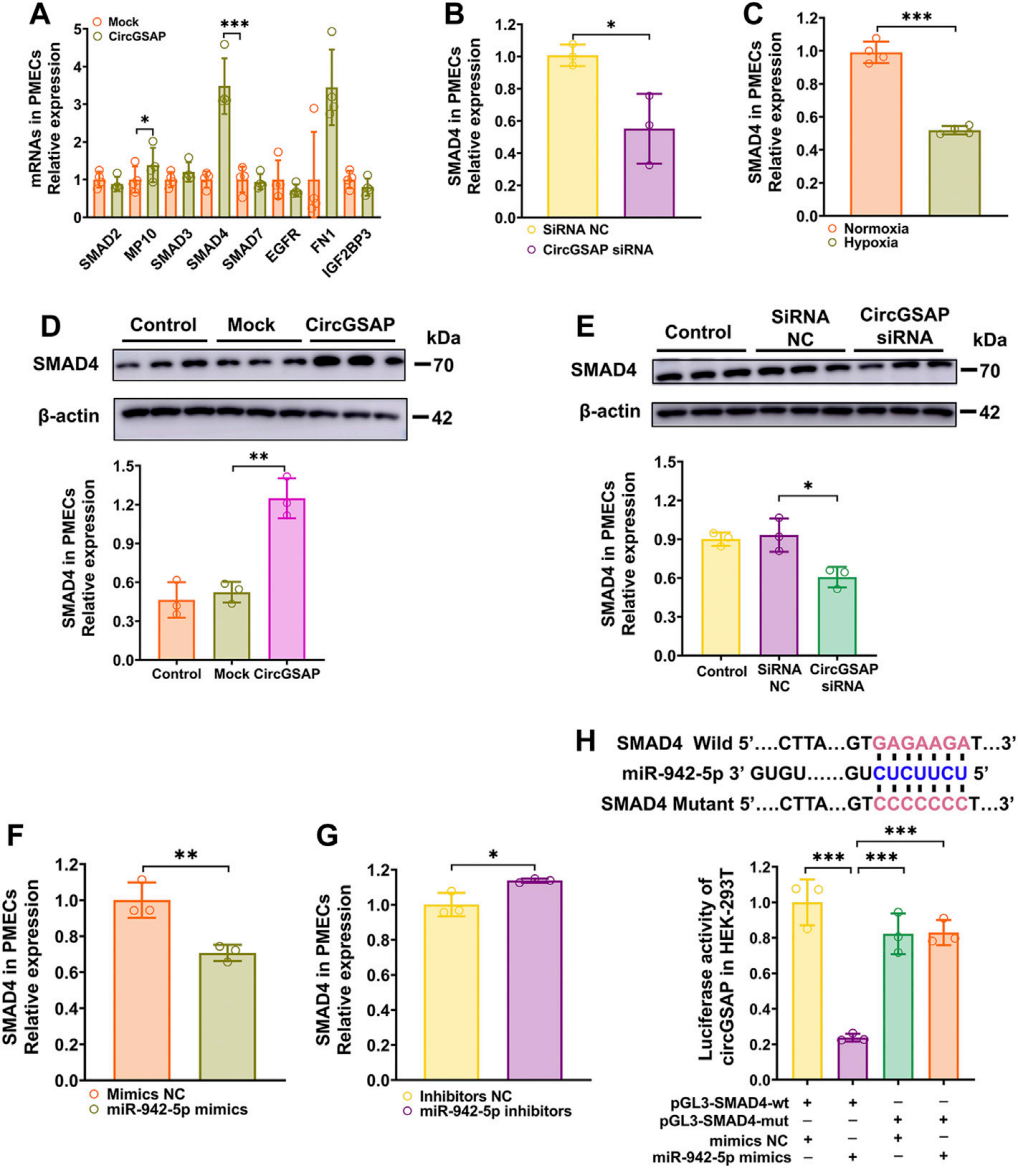
SMAD4 is The Target of miR-942-5p and is regulated by circGSAP. **(A**,**B)** Expression levels of SMAD4 in PMECs treated with circGSAP plasmid and circGSAP siRNA performed by qRT-PCR (*n* = 3). **(C)** Expression levels of SMAD4 in hypoxia treated PMECs (*n* = 4). **(D**,**E)** Expression levels of SMAD4 in PMECs treated with circGSAP plasmid and circGSAP siRNA performed by WB (*n* = 3). **(F**,**G)** Expression levels of SMAD4 in PMECs treated with miR-942-5p mimics and inhibitor (*n* = 3). **(H)** TargetScan predicted binding sites of miR-942-5p and SMAD4, and dual luciferase assays were used to validate the interactions between miR-942-5p and SMAD4 (*n* = 3). All data are presented as the mean ± SEM. *p < 0.05; **p < 0.01; ***p < 0.001.

Targets of miR-942-5p were predicted by two bioinformatic algorithms (miRWalk and TargetScan). Those related to cell proliferation, apoptosis and cell cycle were selected and then detected by qRT-PCR (Figure 5A). The results showed that the expression of SMAD4, a key transcription factor diminished in hypoxic PMECs (Figure 5C) and involved in transforming growth factor-β (TGF-β) signaling (Ezrova et al., 2021), as well as cell cycle signaling, was significantly upregulated in PMECs after overexpression of circGSAP (Figures 5A,D) and was decreased in PMECs following silenced circGSAP (Figures 5B,E), implying that SMAD4 expression is regulated by circGSAP. In addition, SMAD4 level was also expression affected by miR-942-5p (Figures 5F,G). To verify whether SMAD4 mRNA is targeted by miR-942-5p, a dual luciferase reporter assay was performed using 3′ UTR sequence fragments, containing the SMAD4 predicted target region (wt) of miR-942-5p and a mutated target region (mut) inserted downstream of a luciferase reporter (Figure 5H). Cell co-transfection with miR-942-5p mimics and SMAD4-wt showed a significant reduction in firefly luciferase reporter activity compared with that in the negative control group, and the SMAD4-mut groups showed no notable changes (Figure 5H). This finding confirmed the direct binding interaction between miR-942-5p and SMAD4.

### SMAD4 regulate the over-proliferation, G1/S transition and apoptosis resistant of PMECs

**FIGURE 6:**
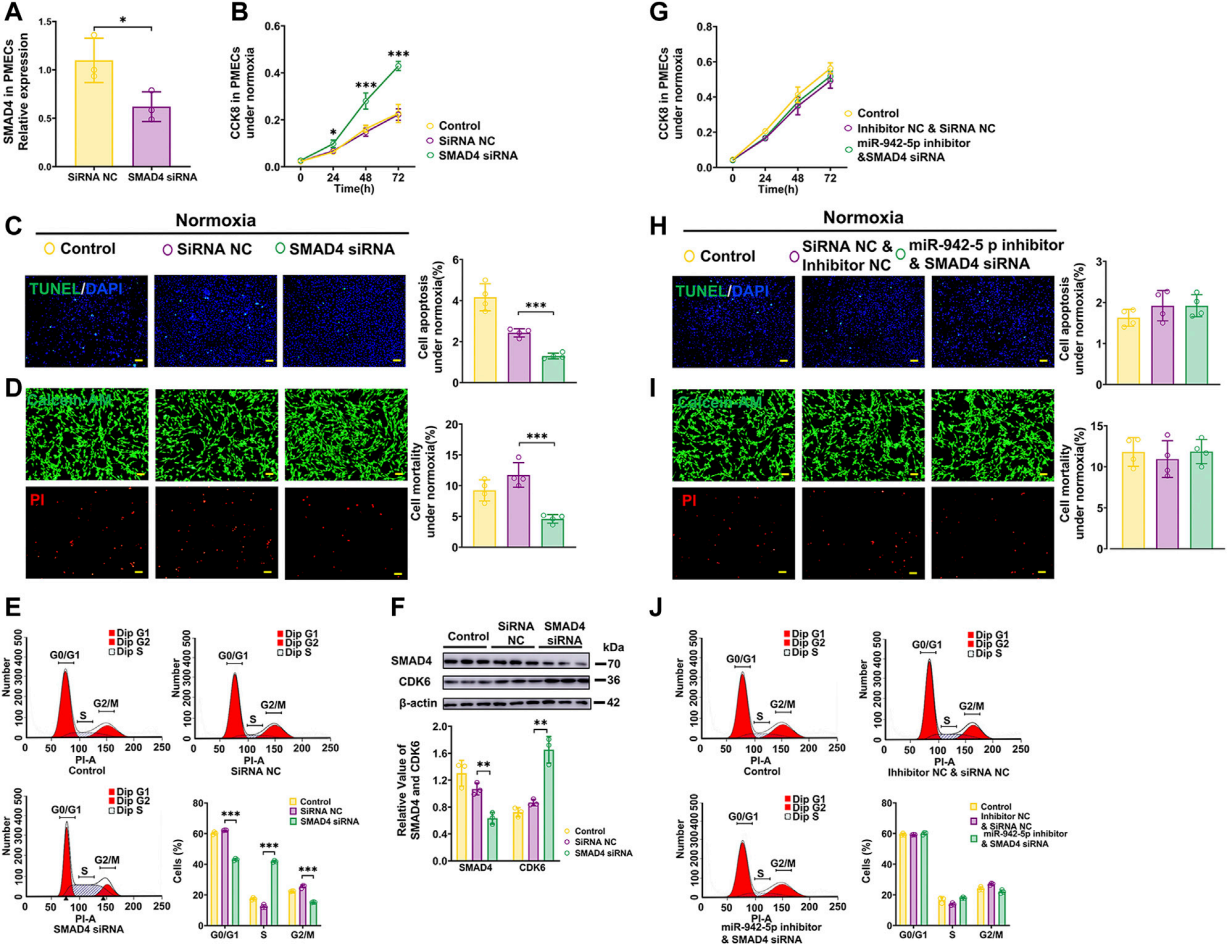
Effects of SMAD4 on the proliferation, apoptosis and cell cycle of PMECs. **(A)** Expression levels of SMAD4 in PMECs treated with SMAD4 siRNA (*n* = 3). **(B**–**E)** Cell proliferation analysis, apoptosis analysis, mortality analysis (*n* = 4) and cell cycle analysis (*n* = 3) of PMECs with transfecting SMAD4 siRNA under normoxia. **(F)** The expression of CDK6 in PMECs transfected with SMAD4 siRNA was analyzed by WB (*n* = 3). **(G**–**J)** Cell proliferation analysis, apoptosis analysis, mortality analysis (*n* = 4) and cell cycle analysis (*n* = 3) of PMECs with co-transfecting miR-942-5p inhibitor and SMAD4 siRNA under normoxia. All data are presented as the mean ± SEM. (scale bar, 100 μm). *p < 0.05; **p < 0.01; ***p < 0.001.

Next, the data demonstrated that SMAD4 siRNAs (Figure 6A) effectively promote proliferation, apoptosis resistance, and G1/S transition of PMECs (Figures 6B–E). and enhanced the expression of CDK6 (Figure 6F). While cells treated with SMAD4 siRNAs in the presence of miR-942-5p inhibitor could attenuate those observed impacts of miR-942-5p on proliferation, apoptosis, as well as cell cycle under normoxic conditions (Figures 6G–J). These results demonstrate that miR-942-5p promotes proliferation and G1/S transition, and apoptosis resistant in PMECs by targeting SMAD4.

### CircGSAP/miR-942-5p/SMAD4 pathway effect the proliferation, apoptosis, and cell cycle on PMECs

**FIGURE 7:**
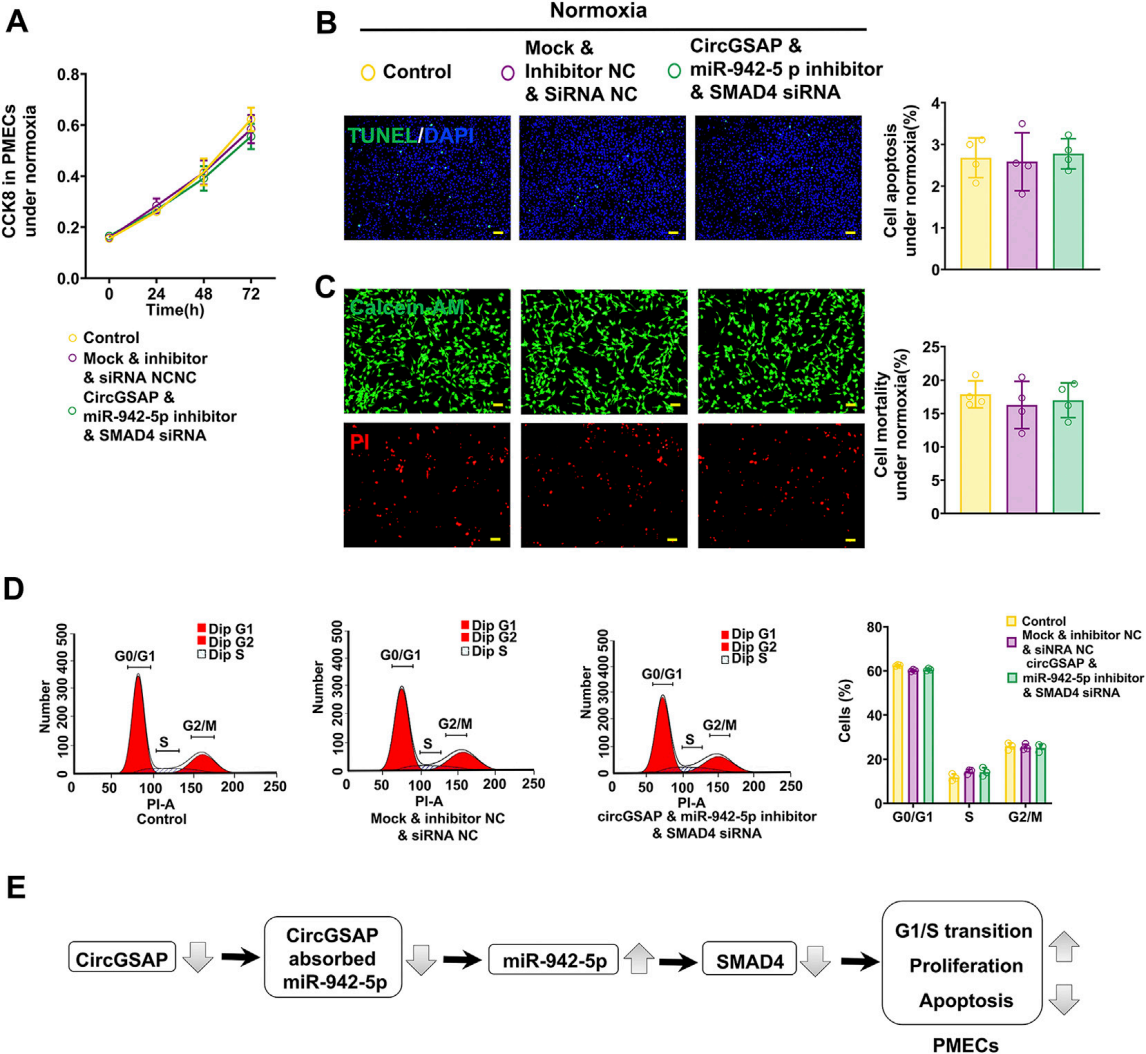
CircGSAP/miR-942-5p/SMAD4 pathway effect the proliferation, apoptosis, and cell cycle on PMECs. **(A**–**D)** Cell proliferation analysis, apoptosis analysis, mortality analysis (*n* = 4) and cell cycle analysis (*n* = 3) of PMECs with co-transfecting circGSAP, miR-942-5p inhibitor and SMAD4 siRNA under normoxia. **(E)** A schematic diagram to illustrate the hypothetical model. All data are presented as the mean ± SEM. (scale bar, 100 μm). *p < 0.05; **p < 0.01; ***p < 0.001.

The above results have shown that the levels of SMAD4 are regulated by circGSAP and miR-942-5p, and overexpression circGSAP and silencing miR-942-5p promoted the proliferation, apoptosis resistance and cell cycle change of PMECs. We next observed the effect of the co-transfection of circGSAP, miR-942-5p inhibitor and SMAD4 siRNA on PMECs, and found that there were no significant differences in cell proliferation, apoptosis and cell cycle between the co-transfection group and the control group or NC group (Figures 7A–D). The results indicated that SMAD4 siRNA could partially reverse the changes of PMECs functions induced by circGSAP together with miR-942-5p inhibitor, which further confirmed that the role of circGSAP/miR-942-5p to regulate PMECs through SMAD4.

## Discussion

In the present study, we found that circGSAP is downregulated in hypoxia-induced PMECs and lung tissues of patients with COPD-PH. Further validation experiments demonstrated that circGSAP was mainly expressed in the cytoplasm of PMECs. Loss-of-function studies using siRNA and gain-of-function studies using plasmid expression suggested the critical roles of low-circGSAP in PMECs dysfunction *via* enhancement of cell growth, apoptosis resistance, and G1/S cell cycle phase transition in PMECs. Mechanistically, circGSAP functioned as miR-942-5p sponge and interacted with *SMAD4* and CDK6 to inhibit cell growth, apoptosis resistance, and G1/S transition.

Some studies have evaluated circRNAs in PH, and these studies demonstrated that circRNA levels generally increase or decrease in different types of PH, and revealed the functions of circRNAs as miRNA sponges and biomarkers (Zhang et al., 2019; Ma et al., 2020; Su et al., 2020; Zhang et al., 2020; Jiang et al., 2021). Our previous study showed that lower circGSAP levels of peripheral blood mononuclear cells may be an emerging biomarker for the diagnosis and prognosis of IPAH (Yuan et al., 2021). In the present study, we found that circGSAP was downregulated in lung tissues of patients with COPD-PH. We further found that circGSAP was mainly expressed in the cytoplasm of PMECs, supporting the likelihood that circGSAP may regulate corresponding pathophysiological processes by acting as miRNA sponge.

PH has emerged as a pulmonary vascular disease with severe PMECs dysfunction. Endothelial dysfunction manifests with features such as, as a cancer cell-like phenotype including unchecked proliferation and apoptosis resistance (Denekamp and Hobson, 1982; Yu and Chan, 2017). The investigation of non-coding RNA is an emerging field in endothelial dysfunction and vascular biology (Hulshoff et al., 2019), including non-coding RNAs such as circRNAs, miRNAs, and long coding RNAs (lncRNAs). Michalik et al. showed that the lncRNA MALAT1 is upregulated in hypoxia and regulated endothelial cell function and vessel growth (Michalik et al., 2014). However, there are few studies on the effect of circRNAs on PMECs. In the present study, we showed that circGSAP could alleviate hypoxia-induced PMECs over-proliferation and apoptosis resistance, and inhibit PMECs G0/G1 to S phase transition. These results are consistent with our previous studies on circGSAP (Yuan et al., 2021), and indicate that circGSAP modulates PMECs function and is involved in PH.

CircRNA acts as miRNA sponge to inhibit the expression of target proteins. We found that circGSAP binds to miR-942-5p using a combination of bioinformatics analysis and whole transcriptome sequencing. Then, using FISH, we observed colocalization between circGSAP and miR-942-5p in the cytoplasm of PMECs. Additionally, dual luciferase assays showed that circGSAP binds to miR-942-5p. More importantly, we verified that miR-942-5p is involved in regulating the proliferation and apoptosis of PMECs. Many studies have shown that miR-942-5p regulates cell proliferation and disease progression in different types of tumors (Li et al., 2020; Wang et al., 2020; Gu et al., 2021; Zhang et al., 2021); these findings are consistent with our results, because PMECs dysfunction in PH is a cancer cell-like phenotype. However, Wang et al. found that miR-942-5p could inhibit PASMCs proliferation *in vitro* (Wang et al., 2021), which is inconsistent with our results; a possible reason for this is the difference in the cell types studied and the cell culture conditions. Evaluating the impact of miR-942-5p on PASMCs during PH development may also help clarify the basis for this difference.

We found that *SMAD4* is a target of miR-942-5p using bioinformatics software analysis. *SMAD4* is a downstream mediator of the transforming growth factor-β (TGF-β) signaling superfamily, regulating cell proliferation, apoptosis, and cell cycle in PH (Morikawa et al., 2016; Guignabert and Humbert, 2021). Zakrzewicz et al. found that expression of two key TGF-β intracellular signaling molecules, *SMAD3* and *SMAD4*, was decreased in PH rats. Such dysregulated transforming growth factor-β signaling may have implications for the activation state of the endothelium (Zakrzewicz et al., 2007). In addition, loss of *SMAD4* is associated with disease progression and poor prognosis in many tumorigenic diseases (Ogawa et al., 2019; Liang et al., 2020; Ezrova et al., 2021), and loss of *SMAD4* causes cardiac dysfunction and dilated cardiomyopathy (Umbarkar et al., 2019). However, it has been unclear whether circGSAP regulates PMECs proliferation, apoptosis, and cell cycle through the target gene *SMAD4* of miR-942-5p. We found that *SMAD4* was downregulated in hypoxia induced PMECs, and *SMAD4* knockdown promoted PMECs proliferation and apoptosis resistance. Dual luciferase assays demonstrated that *SMAD4* was a target of miR-942-5p. Taken together, our results show that the circGSAP/miR-942-5p/S*MAD4* pathway regulates hypoxia-induced PMECs proliferation and apoptosis.

Interestingly, we also found that circGSAP knockdown promoted G1/S transition. The deregulation of cell cycle progression, particularly the G1/S transition, is a common feature of cell proliferation, and targeting cell cycle pathways has been considered a promising strategy for cancer therapy (Otto and Sicinski, 2017). Cyclins and cyclin-dependent kinases (CDKs) are two classes of regulators of cell cycle progression. Transition to the next cell cycle phase depends on the activity of the CDK-cyclin complex, which is frequently deregulated in cancer cells, leading to persistently elevated levels of active cyclin-CDK complexes, and thus uncontrolled cell growth (Roskoski, 2016; Weiss et al., 2019). CDK6 is the key driver of G1/S transition and cell division, and its expression and activity are tightly controlled (Bertoli et al., 2013). Here, we identified circGSAP as a novel CDK6 regulator, promoting G1/S transition and PMECs proliferation.

The present study has several limitations, such as the small number of lung tissue samples from COPD-PH patients. While the other experiments in the study confirmed the potential effect of circGSAP in IPAH, better stratification, larger sample size, and multicenter studies are needed to further verify these results. Second, even though we found that circGSAP was downregulated in MCT-induced PH rats and SU5416-treated hypoxia-induced PH rats (Yuan et al., 2021), more *in vivo* experiments are needed to elucidate the details, which will be one of the goals of our future studies. In addition, although we found that circGSAP regulates the expression of CDK6 and thus influences G1/S transition, the specific mechanism has not been explored and we will continue to address this in future studies. Last, our localization experiments show that circGSAP can also be expressed in PASMCs or inflammatory cells around blood vessels. Whether this affects the function of PASMCs or participates in immune or inflammatory regulation in PH pathogenesis needs further exploration, to arrive at a comprehensive understanding of the underlying mechanism of action of circGSAP in alleviation of PH progression.

## Conclusions

Our findings demonstrate that circGSAP is downregulated in lung tissues of patients with COPD-PH and in hypoxia-induced PMECs, resulting in increased cell proliferation, apoptosis resistance and G1/S transition. Downregulation of circGSAP acts as miR-942-5p sponge, thereby reducing SMAD4 expression, ultimately leading to endothelial injury. Therefore, we identified and elucidated the function and mechanism of circGSAP in PMECs, which may provide a novel approach for developing effective therapeutic interventions for PH patients.

## Data Availability

The raw data supporting the conclusions of this article will be made available by the corresponding authors, without undue reservation.
