# Supplementary material for: CircGSAP regulates the cell cycle of pulmonary microvascular endothelial cells via the miR-942-5p sponge in pulmonary hypertension
Source: Front Cell Dev Biol. 2022 Aug 11;10:967708. doi: 10.3389/fcell.2022.967708 (PMC9428790; doi:10.3389/fcell.2022.967708)
Supplement: Supplementary file 1 [file Table1.DOCX]

**CircGSAP regulates the cell cycle of pulmonary microvascular endothelial cells via the miR-942-5p sponge in pulmonary hypertension**

***Yuanyuan Sun*** ***^1,2†^, Wenhui Wu ^1†^, Qinhua Zhao ^1†^, Rong Jiang ^1^, Jinling Li ^1^, Lan Wang ^1^,*** ***Shijin Xia ^3^, Mingjie Liu ^4^, Sugang Gong ^1^, Jinming Liu ^1*^, Ping Yuan ^1*^***

**Online data supplement**

Table S1

Figure S1

Figure S2

Figure S3

| **Table S1. Primers, siRNA and mimics or inhibitors used in this study** | |
| --- | --- |
| **qRT-PCR** | |
| circGSAP | F: ACAGAAGACATTGCATTGCCT |
|  | R: AGAGATTGTGGCAGATCAGGT |
| GSAP | F: GCTACCTCATTGCCCTTTACAG |
|  | R: ACTTGTCTCTGAATATACACTCCAG |
| GAPDH | F: TCTCTGCTCCTCCTGTTCGA |
|  | R: GCGCCCAATACGACCAAATC |
| miR-942-5p | F: ACACTCCAGCTGGGTCTTCTCTGTTTTGGC |
|  | R: GTGCAGGGTCCGAGGT |
| U6 | F: TGCTTCGGCAGCACATATAC |
|  | R: TCACGAATTTGCGTGTCATC |
| SMAD4 | F: GCCCAGGATCAGTAGGTGGA |
|  | R: GGTCCCCAGCCTTTCACAAA |
| CDK6 | F: CCCCTCAGGTGCAATGATTC |
|  | R: ATCCCTCCTCTTCCCTCCTC |
| miR-891a-5p | F：ACACTCCAGCTGGGTGCAACGAACCTGAGC |
|  | R：GTGCAGGGTCCGAGGT |
| miR-103a-2-5p | F：ACACTCCAGCTGGGAGCTTCTTTACAGTGCT |
|  | R：GTGCAGGGTCCGAGGT |
| miR-106a-3p | F：ACACTCCAGCTGGGCTGCAATGTAAGCACT |
|  | R：GTGCAGGGTCCGAGGT |
| miR-98-5p | F：ACACTCCAGCTGGGTGAGGTAGTAAGTTGT |
|  | R：GTGCAGGGTCCGAGGT |
| miR-148a-5p | F：ACACTCCAGCTGGGTCAGTGCACTACAGAA |
|  | R：GTGCAGGGTCCGAGGT |
| miR-206 | F：ACACTCCAGCTGGGTGGAATGTAAGGAAGT |
|  | R：GTGCAGGGTCCGAGGT |
| miR-298 | F: ACACTCCAGCTGGGAGCAGAAGCAGGGAGGTT |
| miR-298 | R: GTGCAGGGTCCGAGGT |
| miR-30a-5p | F：ACACTCCAGCTGGGTGTAAACATCCTCGAC |
|  | R：GTGCAGGGTCCGAGGT |
| miR-504-5p | F：ACACTCCAGCTGGGAGACCCTGGTCTGCAC |
|  | R：GTGCAGGGTCCGAGGT |
| BMP10 | F：TTGCAACAGATCGGACCTCC |
|  | R：GGACACATTGAAGAGGAGGGG |
| SMAD2 | F：GAAAGGGTGGGGAGCAGAAT |
|  | R：CAACGCACTGAAGGGGATCC |
| SMAD3 | F：CAGTTGACCCGAATGTGCAC |
|  | R：TCAGGTGCAGCTCAATCCAG |
| SMAD7 | F：TGACTGTCCAGATGCTGTGC |
|  | R：ATCCCCAGGCTCCAGAAGAA |
| EGFR | F：CGAGTACCTCATCCCACAGC |
|  | R：CACGGTGGAATTGTTGCTGG |
| FN1 | F：GGTGAACCCAGTCCCGAAG |
|  | R：CGGGAATCTTCTCTGTCAGCC |
| IGF2BP3 | F：TCTATGCTTGCCAGGTTGCC |
|  | R：CCGTCTTGACTGAGGTGGTC |
| Rat GAPDH | F: AAGATGGTGAAGGTCGGTGT |
|  | R: TGACTGTGCCGTTGAACTTG |
| Rat CDK6 | F: TGTTTCAGCTTCTCCGAGGT |
|  | R: CGAGGTAAGGGCCATCTGAA |
| **SiRNAs sequences:** | |
| si-circGSAP #1 | Sense (5' to 3') UGCCUCUUAUGAAGACUAUTT  Antisense (5' to 3') AUAGUCUUCAUAAGAGGCATT |
| si-circGSAP #2 | Sense (5' to 3') UUAUGAAGACUAUUAUGUGTT  Antisense (5' to 3') CACAUAAUAGUCUUCAUAATT |
| si-circGSAP #3 | Sense (5' to 3') AUGAAGACUAUUAUGUGGCTT  Antisense (5' to 3') GCCACAUAAUAGUCUUCAUTT |
| si-SMAD4 #1 | Sense (5' to 3') CCAGCAUCCACCAAGUAAUTT  Antisense (5' to 3') AUUACUUGGUGGAUGCUGGTT |
| si-SMAD4 #2 | Sense (5' to 3') GGUGGAGAGAGUGAAACAUTT  Antisense (5' to 3') AUGUUUCACUCUCUCCACCTT |
| si-SMAD4 #3 | Sense (5' to 3') GCCUCCCAUUUCCAAUCAUTT  Antisense (5' to 3') AUGAUUGGAAAUGGGAGGCTT |
| **PCR primers for plasmids construction** | |
| circGSAP | F: CGGAATTCTAATACTTTCAGACTATTATGTGGCTGTTTACT |
|  | R: CGGGATCCAGTTGTTCTTACTTCATAAGAGGCAATGCAATG |
| **Mimics and inhibitor sequences** | |
| miR-942-5p mimics | Sense (5' to 3') UCUUCUCUGUUUUGGCCAUGUG |
|  | Antisense (5' to 3') CAUGGCCAAAACAGAGAAGAUU |
| miR-942-5p inhibitor | CACAUGGCCAAAACAGAGAAGA |
| **Primers for dual luciferase activity reporter system** | |
| circGSAP-WT | F: GGGCCCGGGTCCCTGGTATTGGATTGTTG |
|  | R: GAATTCGGCAGGCAGAGACATTCTCAG |
| circGSAP-MUT | F: CTTAGACTGTCCCCCCCTGGCTGCGTTGCACTGCGCGCTCTA |
|  | R: AACGCAGCCAGGGGGGGACAGTCTAAGCAAGTGTTCTGCAGAA |
| SMAD4-WT | F: GGGCCCGAACTTGTCAGGCATGGCTC |
|  | R: GAATTCCAACCAACCTTGTGCCTAGA |
| SMAD4-MUT | F: ATTTTAAAGGCCCCCCCCTTCTCAAAGTTAATTCACCTATGTTATT  TTGTGTAC |
|  | R: CTTTGAGAAGGGGGGGGCCTTTAAAATCAGCAATACTGGATTAT  TCTGTAG |
| **Sequences for probe** | |
| circGSAP | 5’Cy3-CACATAATAGTTTCATAAGAGGCA- 3’Cy3 |
| miR-942-5p | 5'FAM-CACATGGCCAAAACAGAGAAGA |

**Supplementary Figure legends**

**Figure S1****.** The map and sequencing of circGSAP **(A)** The map of circGSAP. **(B)** Sequence of linear circGSAP was identified by Sanger sequencing.

**Figure S2.** Effects of circGSAP on the proliferation, apoptosis and cell cycle of PMECs under normoxia. **(A)** Expression levels of circGSAP in PMECs transfection with circGSAP plasmid under normoxia. **(B-E)** Cell proliferation analysis, EDU analysis, cell apoptosis analysis and cell cycle analysis of PMECs with overexpressing circGSAP under normoxia. All data are presented as the mean ± SEM. (scale bar, 100 μm). **p* < 0.05; ***p* < 0.01; ****p* < 0.001.

**Figure S3.** Effects of miR-942-5p on the proliferation, apoptosis and cell cycle of PMECs under normoxia. **(A)** Expression levels of miR-942-5p in PMECs treated with miR-942-5p inhibitor under normoxia. **(B-F)** Cell proliferation analysis, apoptosis analysis, mortality analysis and cell cycle analysis of PMECs with miR-942-5p inhibitor under normoxia. All data are presented as the mean ± SEM. (scale bar, 100 μm). **p* < 0.05; ***p* < 0.01; ****p* < 0.001.
