# Supplementary figures and images for: CircGSAP regulates the cell cycle of pulmonary microvascular endothelial cells via the miR-942-5p sponge in pulmonary hypertension
Source: Front Cell Dev Biol. 2022 Aug 11;10:967708. doi: 10.3389/fcell.2022.967708 (PMC9428790; doi:10.3389/fcell.2022.967708)

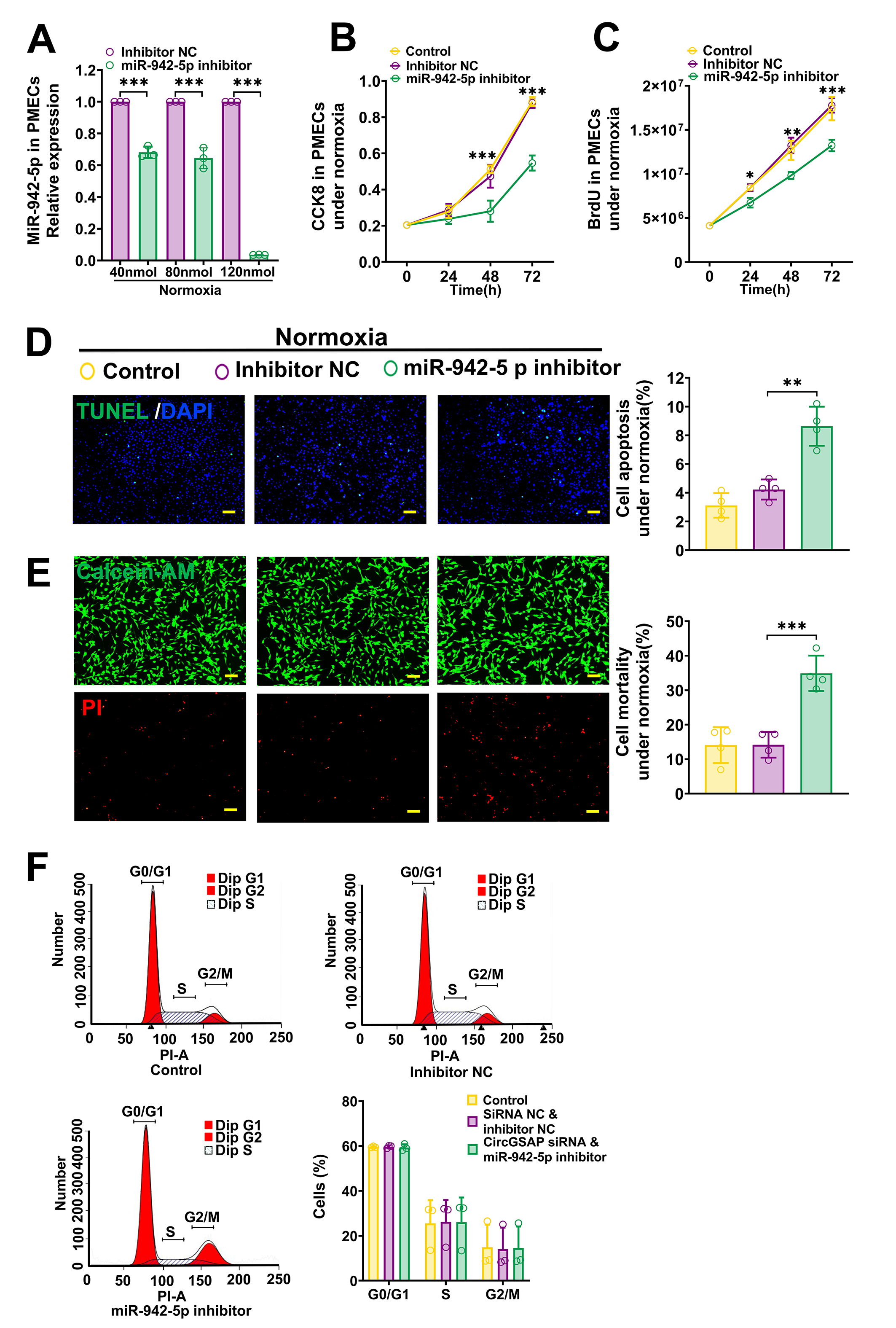

Supplement: Supplementary file 2 [file Image3.tif]

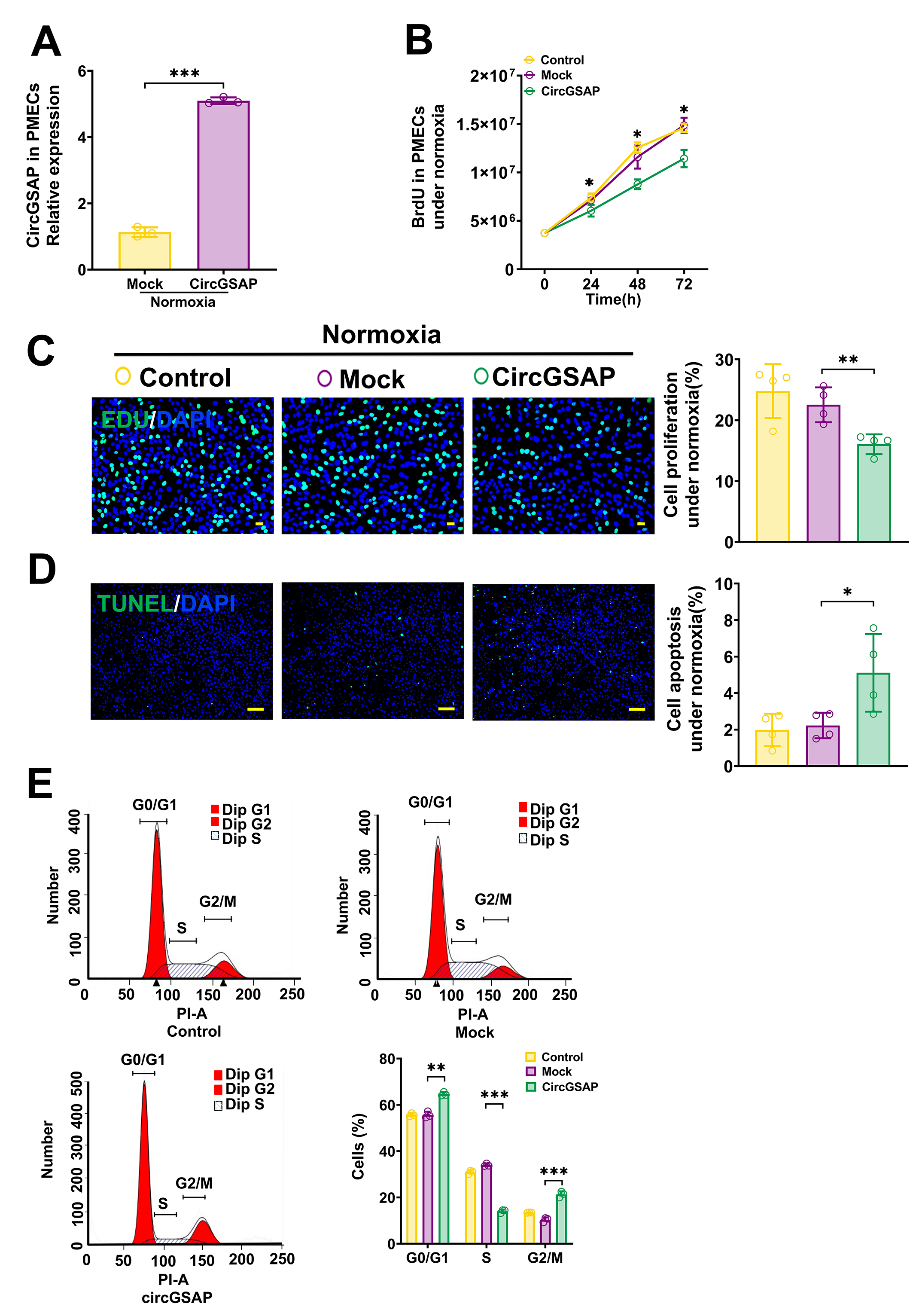

Supplement: Supplementary file 3 [file Image2.tif]

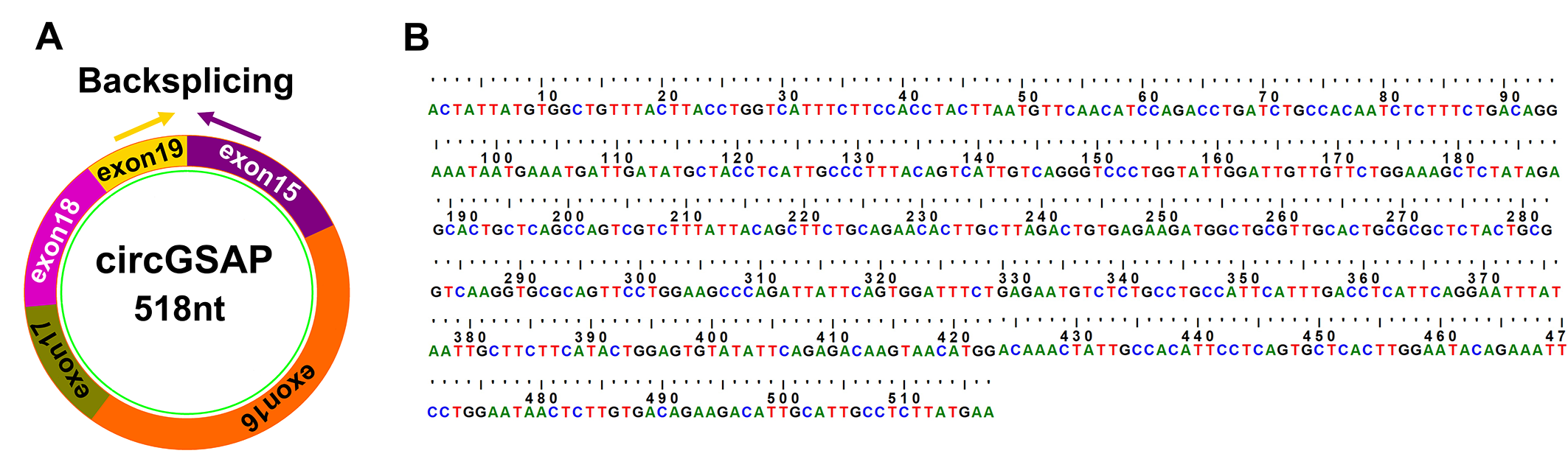

Supplement: Supplementary file 4 [file Image1.tif]
